# Supplementary material for: Structural Basis for the Interaction between the Ezrin FERM-Domain and Human Aquaporins
Source: Int J Mol Sci. 2024 Jul 12;25(14):7672. doi: 10.3390/ijms25147672 (PMC11277499; doi:10.3390/ijms25147672)
Supplement: Supplementary file 1 [file ijms-25-07672-s001.zip › ijms-3092202-supplementary.pdf]

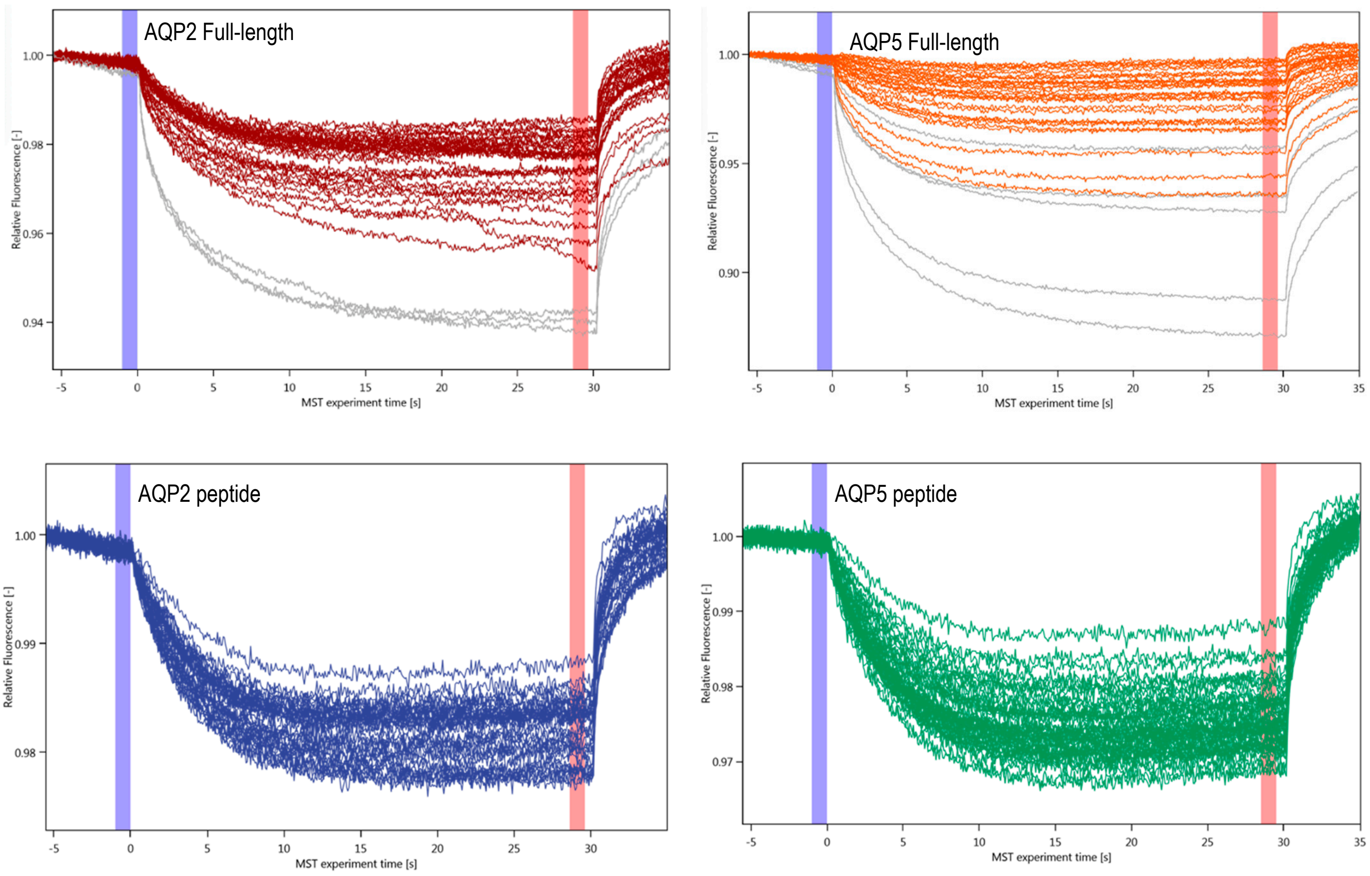

Figure S1. Microscale thermophoresis. MST traces for the interaction between the full-length AQP2 (red) C-terminus AQP2 (blue), full-length AQP5 (orange) and C-terminus AQP5 (green) C-termini and the human ezrin FERM-domain. Each trace corresponded to one capillary with a different concentration of AQP2/5 construct whereas the concentration of FERM is constant. The columns correspond to the fluorescence values before (blue) and after (red) heating that are used to calculate ΔF_{norm}. Grey traces were omitted due to deviations in initial fluorescence values.

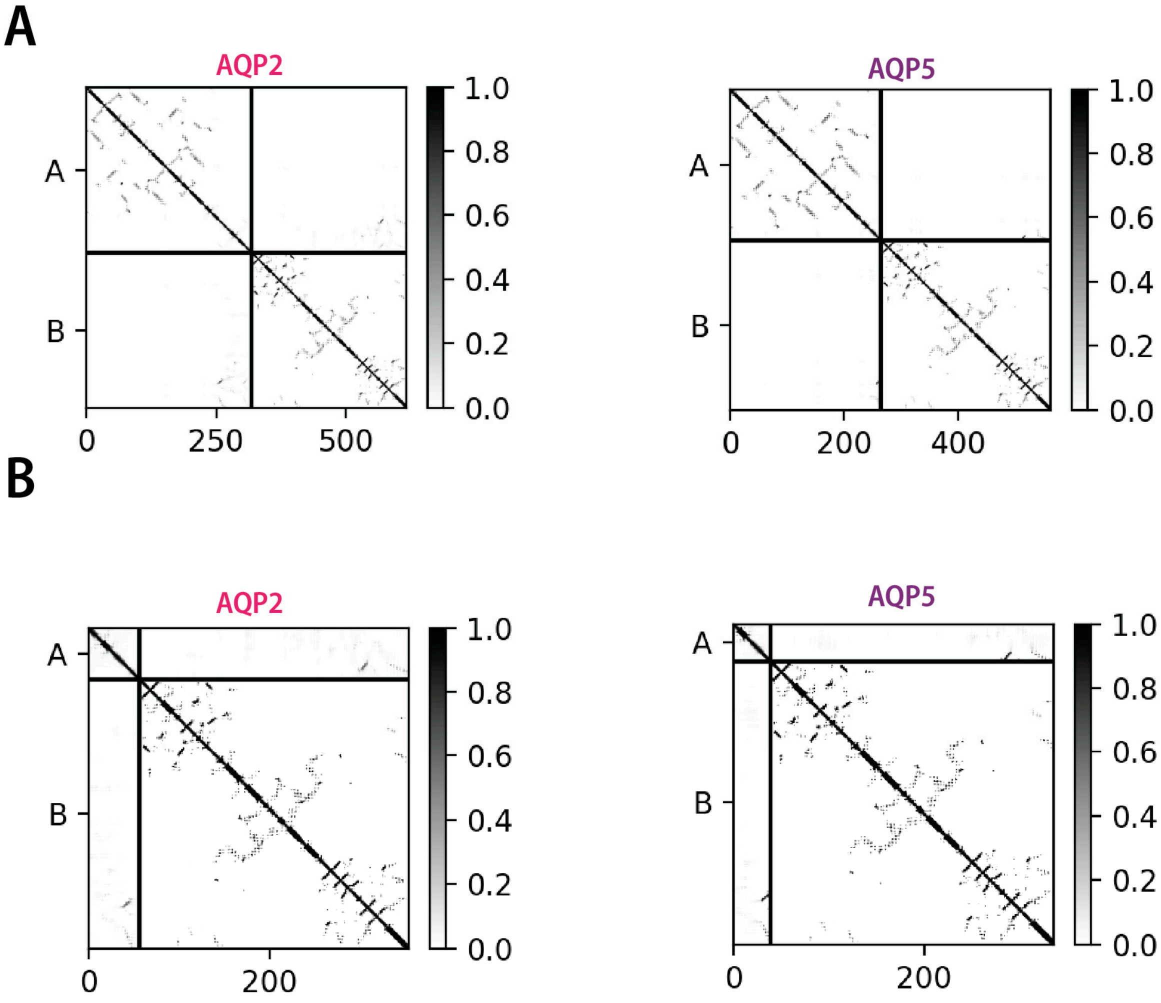

Figure S2. In silico modelling. Probability plots from ColabFold showing the likelihood of the predicted contacts in the complexes. In each plot, A corresponds to the aquaporin and B to FERM. (**A**) Full length AQP2 and AQP5 docked with Ezrin-FERM. (**B**) C-terminal domains of AQP2 and AQP5 docked with Ezrin-FERM.
